# A Rare Case of Primary Anorectal Hodgkin Lymphoma in an HIV-Positive Patient: A Case Report and Literature Review

**DOI:** 10.7759/cureus.38796

**Published:** 2023-05-09

**Authors:** Fidel M Perez, Gladys L Valdez, Patrick WU, Moazzum Bajwa, Faheem Jukaku

**Affiliations:** 1 Family Medicine, Riverside University Health System Medical Center, Moreno Valley, USA; 2 Family Medicine, Innercare Coachella, Coachella, USA; 3 Infectious Disease, Riverside University Health System Medical Center, Moreno Valley, USA

**Keywords:** anorectal lymphoma, anal hodgkin lymphoma, epstein-barr virus and hiv, ebv-eber ish, hodgkin and reed/stemberg (hrs) cells, aids-related lymphoma, colorectal lymphomas, hodgkin lymphoma, ebv, hiv/aids

## Abstract

Lymphoma is a well-known complication related to HIV infection; of these, non-Hodgkin lymphoma (NHL) is the most common subtype with Hodgkin lymphoma (HL) occurring less frequently. We present a rare case of a 35-year-old male with a history of HIV/AIDS well-controlled on antiretroviral therapy (ART) with an atypical HL presentation. He arrived at the emergency department with rectal bleeding, 30-pound unintentional weight loss, and subjective fever. CT scan of the abdomen and pelvis showed a circumferential mass extending from the mid-rectum to the anus, with extensive local lymphadenopathy. He underwent multiple biopsies of the mass and adjacent lymph nodes. The pathology report showed EBV-positive lymphoma with features of classical Hodgkin lymphoma (cHL) (positive for EBV-EBER by in-situ hybridization). He was started on A+AVD (brentuximab plus doxorubicin, vinblastine and dacarbazine). The patient tolerated the chemotherapy well without significant complications. We want to encourage physicians and providers to include anorectal HL in their differential diagnosis for HIV/AIDS patients with atypical rectal malignancy presentations and subsequent reporting of these cases.

## Introduction

A group of aggressive lymphomas known as acquired immunodeficiency syndrome (AIDS)-related lymphomas are highly indicative of HIV/AIDS; most of these are non-Hodgkin lymphoma (NHL). These lymphomas can have an extra-nodal location, including the involvement of the gastrointestinal tract. However, gastrointestinal lymphomas are uncommon and constitute about 1-4% of all gastrointestinal malignancies [1], and even fewer subtypes involve the colorectal region. Primary colorectal lymphomas represent 0.2% of all gastrointestinal lymphomas, whereas primary Hodgkin lymphoma (HL) is usually present in 1-3% of the totality of these cases [1-3]. An extensive report series of gastrointestinal lymphoma and Hodgkin's disease correlation demonstrated that the incidence of primary rectal HL was one out of 1,423 cases [4]. Most of these occurrences are found in HIV-positive individuals or associated with inflammatory bowel disease (IBD) [1, 3]. 

Here we present a rare case of primary rectal lymphoma in a patient with advanced HIV/AIDS. 

## Case presentation

Our patient was a 35-year-old male of Hispanic ethnicity, sexually active exclusively with male partners, admitting to being engaged in a promiscuous lifestyle, with a history of HIV/AIDS for seven years; initially reported intermittent compliance to antiretroviral therapy (ART), used elvitegravir/cobicistat/emtricitabine/tenofovir alafenamide for one year without issues in 2017, but for unclear reasons stopped the ART regimen between 2017-2019; in 2019 he was started on bictegravir/emtricitabine/tenofovir alafenamide, and had been compliant ever since. His viral load was 630,000 copies/mL in 2019 when he established medical care, and one year after, his viral load showed 63 copies/mL; during current admission, his viral load reported 24 copies/mL. His CD4% was 11% on admission time, similar to 7% and 10% on years prior on two different occasions. Also, he had a history of syphilis, HPV (human papillomavirus) E6/E7+ with anal condyloma acuminata, and substance abuse (methamphetamines, tobacco). During questioning on admission, no previous history of opportunistic infections or family history of malignancy was reported. 

The patient presented to the emergency department with painless rectal bleeding, 30 lbs unintentional weight loss over two months course, and new associated B-symptoms (fever, persistent fatigue, loss of appetite, night sweats, abdominal fullness, and bloating sensation). Physical examination revealed abdominal tenderness to palpation at the left lower quadrant without palpable masses or organomegaly. Blood assessment presented marked pancytopenia (hemoglobin 6.9 g/dL, white blood cells 2.0 X10e12/L, platelets 26 X10e9/L); plasma Epstein Barr virus (EBV)-PCR reported 17035 copies/mL on admission, 193040 copies/mL one month after admission, and 99563 copies/mL three months after admission.

**Figure 1:**
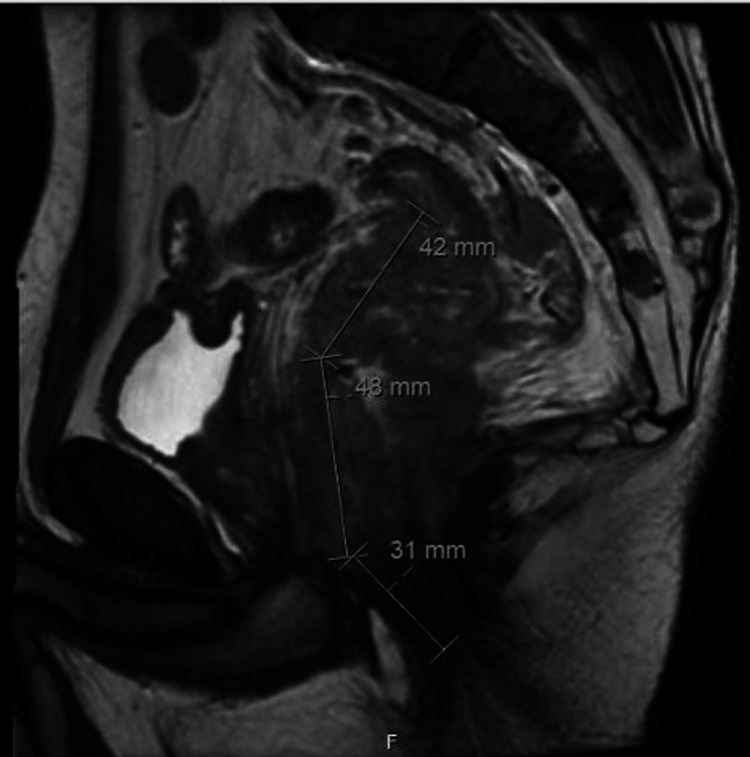
Saggital thin slice, fast-recovery fast spin-echo T2-weighted MRI MRI of the pelvis with contrast: multiplanar T2-weighted, diffusion, and dynamic. Post-gadolinium images were obtained. IMPRESSIONS: (1) Rectal cancer T-Stage=T4b with extension to seminal vesicles, prostate, membranous urethra, and anal sphincter; (2) rectal cancer N-stage= 2b; (3) diffuse marrow signal abnormality of the visualized spine and pelvic bones, may represent marrow hyperplasia secondary to anemia, versus the less likely alternative of diffuse metastatic disease.

**Figure 2:**
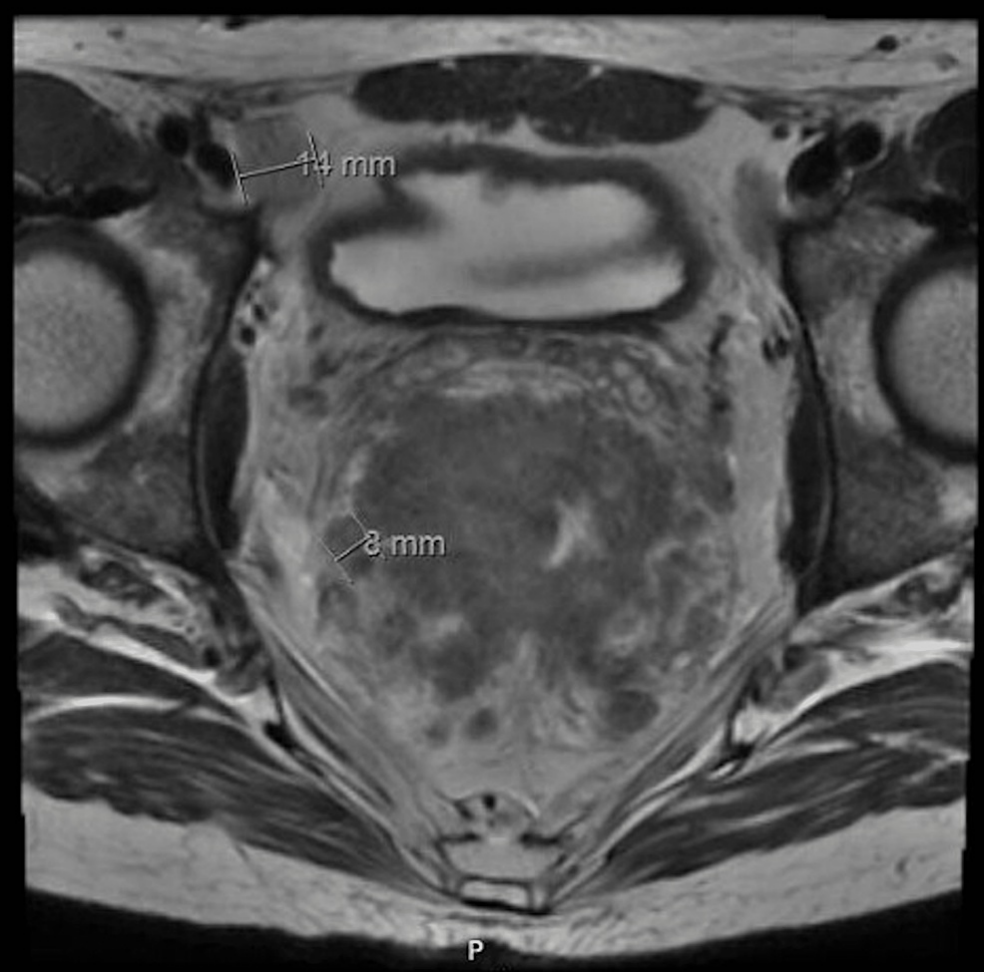
Axial thin slice, fast-recovery fast spin-echo T2-weighted MRI; measurements of two involved lymph nodes

**Figure 3:**
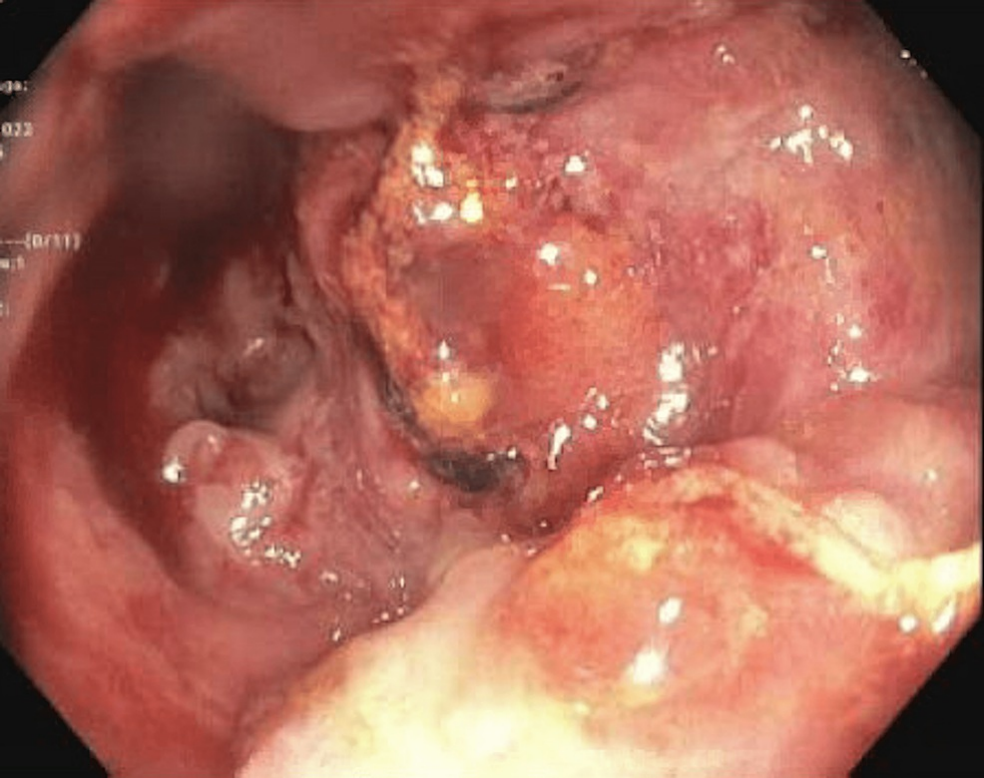
Colonoscopy: rectal view Gastroenterology comments: (1) A fungating, circumferential ulcerated, and infiltrative non-obstructing large mass was found in the rectum, involving distal 10cm of the rectum extending to the anus, biopsied. High suspicion for malignancy; (2) no evidence of large polyps or mass in the rest of the colon, although inadequate prep to identify small or flat polyps.

An MRI scan of the abdomen and pelvis with rectal protocol staged the patient as T4b (extension into the seminal vesicle, prostate, membranous urethra, anal sphincter) N2b (7+ suspicious lymph nodes); imaging identified a circumferential polypoid tumor mass involving the entirety of the rectum, extending from the rectosigmoid junction down to the anal canal, associated with extensive inguinal, iliac, perirectal, inferior mesenteric, and retroperitoneal lymphadenopathy (Figures 1-2). A colonoscopy confirmed the finding of the large rectal mass seen in previous imaging (Figure 3). 

 

Over the subsequent two months, he was discharged and then re-admitted to the hospital on four occasions for worsening initial symptoms; during this time, he underwent multiple biopsies of the mass via flexible sigmoidoscopy and colonoscopy, which showed inflammatory signs but was non-diagnostic for malignancy. Finally, gastroenterology and colorectal surgery performed a joint rectal exam under anesthesia utilizing endoscopic ultrasound to obtain additional biopsies; these specimens were sent out to a specialized pathological center for review, and the final report showed EBV-positive lymphoma with features of classic Hodgkin lymphoma (positive for EBV-EBER (EBV-encoded RNAs) by in-situ hybridization). Confirmatory immunohistochemical studies identified Hodgkin and Reed-Stemberg (HRS) cells, composed of an admixture positive including Ki67, BCL1-2, cMYC, MUM1, EBV/LMP, EBV-EBER ISH (in situ hybridization), and others. A core needle biopsy of the right inguinal lymph node confirmed classical Hodgkin lymphoma. Carcinoembryonic antigen (CEA) levels were 1.3 ng/mL (normal range 0-2.5 ng/mL). The patient initiated a chemotherapy regimen for treatment of HL stage IVB, which includes brentuximab, doxorubicin, vinblastine, and dacarbazine with G-CSF support along with dexrazoxane for cardio-protection; doxorubicin and vinblastine provided at a dose reduced by 50% due to concomitant heart failure with reduced ejection fraction (35-40%) and hyperbilirubinemia. Bleomycin was not given due to ongoing cytopenia. Later, the doxorubicin was dose-escalated to the full dose based on a risk-benefit discussion between the patient and the hematology and oncology team. After completion of four therapy sessions, PET-CT was obtained and showed some residual hypermetabolic activity in the rectum region, but with a significant decrease in tumor bulk and no hypermetabolic adenopathy evident below the diaphragm.

The patient tolerated the aforementioned chemotherapy regimen well without significant complications at the time of this report, 11 months after the initial encounter. 

## Discussion

Primary rectal lymphoma is exceedingly rare, and few detailed cases have been reported. After concluding our literature review, we found 26 cases of primary rectal-anorectal HL that were adequately reported and published; from this amount, seven cases were exclusively anorectal (see Table 1). Unfortunately, other poorly documented cases are too limited for a clinical retrospective study. Hodgkin lymphoma is associated with HIV and EBV infections, both of which were present in the case described in this paper. In many of the reported cases of Hodgkin's lymphoma, the presence of EBV is evident [2], and it is considered a critical factor in the pathogenesis of this malignancy. For instance, in their study of HL cases in HIV, Siebert et al. reported that EBV-encoded RNAs as well as small encoding EBV RNAs in an actively transcribed region of the genome present in latently infected cells, and latent membrane proteins were identified as oncogenic red flags and indicative of latent infections, highlighting that 100% of their cases presented with objective indications of latent EBV infection [5]. Based on this information, immunocompromising illnesses such as HIV infection and medical therapies that induce severe immunodeficiency could allow a foothold for this EBV to promote oncogenicity. Having said that, HIV/AIDS immunodeficiency is an open window for EBV to promote HL. Most of the current reported cases do not offer enough data to know to compare if their immunocompromising condition were under control or not. Regarding predisposing factors, EBV is the infectious agent most frequently associated with HL [6]; however, the incidence of EBV infection appears to vary depending on the population's geographic location and epidemiological characteristics. One Scandinavian study showed that patients with previous EBV infection have a 2.5-fold increased risk of Hodgkin's disease for up to two decades after initial infection [6]. Prior studies have demonstrated EBV infection being present in 30-50% of HL cases in North America and Western Europe and nearly 60% in China, with an evident higher incidence in Latin America, Africa, and Asia [7]. Furthermore, other researchers have found that EBV-positive HL impacts more Hispanic patients regardless of patient age or immune status [8-9]; Chang indicated the high prevalence of EBV-positive HL in Peru (present in 94% of cases) [10].

**Table 1:** Primary Anorectal and Rectal Hodgkin Lymphomas ND: No data, RS: Reed-Stemberg, Tx: Treatment, MOPP: mechlorethamine hydrochloride, Oncovin, procarbazine hydrochloride, and prednisone, ABVD: Adriamycin, bleomycin, vinblastine, and dacarbazine, BV/AVD: Brentuximab / Adriamycin, vinblastine, and dacarbazine, Rad: Radiation, M: Male, F: Female, IS: Immunosuppression, IBD: Inflammatory Bowel Disease, -: Negative, +: Positive

Literature review Clinicopathologic components of anorectal HL								
Source and publication by year	Age/Gender	Location	IS	EBV	Stage/Type	Treatment	Tx outcome	Other information
Warren and Lulenski, 1942 [3]	ND	Rectum	ND	ND	IE	ND	Alive at two years after Tx	
Portman et al., 1954 [3]	ND	Rectum	ND	ND	IE	ND	ND	
Allen et al., 1954 [3]	ND	Rectum	ND	ND	IE	ND	ND	
Warren and Littlefield, 1955 [3]	ND	Rectum	ND	ND	IE	ND	ND	
Dawson et al., 1961 [3]	53/F	Rectum	ND	ND	IE	Surgery and rad	Alive at nine months	
Shapiro, 1961 [3]	46/M	Rectosigmoid	ND	ND	IIE	Mechlorethamine and chlorambucil chemotherapy	Died	
Kuhlmann, 1975 [3]	45/M	Rectosigmoid	ND	ND	IE	ND	ND	
Tabuse, et al 1983 [3]	40/M	Rectum	ND	ND	IE	ND	ND	
Coonley et al., 1984 [4]	33/M	Rectum	HIV+	ND	IIBE	MOPP/ABV	Complete response	No recurrence at eight months post-treatment completion
Picard et al., 1987 [3]	51/M	Rectum	HIV+	+	IE		Unresponsive	Involves liver and mesenteric/retroperitoneal lymph nodes
Pinover et al., 1993 (I) [11]	36/M	Rectum	HIV+	ND	IVb	MOPP/ABV	Unresponsive	Presence of extensive bone marrow involvement; +RS cells and +LeuM1
Pinover et al., 1993 (II) [11]	24/M	Rectum	HIV+	ND	ND	ND	ND	
Marquez-Moreno et al., 1999 [3]	33/M	Anorectal	HIV+	ND	IIBE	ND	ND	+ Regional lymphadenopathy
Sapp et al., 2001 [3]	33/M	Anorectal	HIV+	+	IIE	Local Rad	Deceased at eight months	+ Regional lymphadenopathy
Pagano et al., 2000 [3]	67/M	Rectum	ND	ND	IE	ND	ND	
Simpson et al., 2004 [12]	39/M	Anorectal	HIV+	ND	IVB	ABVD	Alive at two years	+ Bone marrow involvement
Hilman et al., 2005 [9]	37/M	Anorectal	HIV+	+	IIAEX	Failed initial chemotherapy, partially responsive to local radiation	Deceased at 18 months	Metastasized to lungs and liver at six months after initial treatment
Valbuena et al., 2005 [8]	81/M	Rectum	ND	+	IE	Resection	ND	
Bai et al., 2006 [13]	35/M	Rectum	IBD	+	ND	Unspecified chemotherapy	Alive at eight months, with no progression of HL	EBER+
Da Costa et al., 2011 [14]	58/M	Rectum	ND	+	IE	ABVD	Alive at 11 months	Rectosigmoidectomy
Ambrosio et al., 2013 [3]	83/M	Anorectal	ND	+	IE	MOPP	Alive at 20 months	
Rasmussen et al., 2015 [15]	44/M	Rectosigmoid	IBD	+	ND	Resection and ABVD	Alive at four months with almost complete remission	EBER+
Vasudevan et al., 2017 [16]	64/F	Rectum		ND	IE	ABVD	Deceased	
Sarma et al., 2019 [17]	39/F	Anorectal	HIV+	+	ND	ND	Alive at 12 months	EBER+
de Sire et al., 2021 [18]	40/F	Rectum	IBD	+	IVB	ABVD	Alive at 12 months, in clinical remission	EBER+
Present case	35/M	Anorectal	HIV+	+	IVBE	BV / AVD	Alive	EBER+

AIDS-related lymphoma is an infrequent but well-documented complication of HIV infection. In the United States, 3% of patients with HIV/AIDS will present with lymphoma during their lifetime [2]; the rectal-anorectal primary tumor location represents a small subset of this already rare condition. Our experience highlighted the importance of including this condition in our differential diagnosis, as it allowed for the early initiation of appropriate chemotherapy. Advances in oncological treatments over the last 20 years have given patients more chances to succeed and tolerate therapy. For this reason, recognition of early presenting symptoms, including anorectal pain and rectal bleeding, and prompt diagnosis is crucial in affecting similar patients' survival outcomes. 

## Conclusions

The pathophysiology of lymphoma exemplifies the complex interplay between malignancy and immunology in the setting of HIV and EBV coinfection. Herein we presented a patient with HIV/AIDS who was subsequently diagnosed with a rare EBV-positive anorectal HL and underwent chemotherapy successfully. We briefly compare our case to prior satisfactorily reported cases in the literature, hoping to provide valuable scientific data for future researchers. Finally, we want to encourage physicians and providers to include anorectal HL in their differential diagnosis for HIV/AIDS patients with atypical rectal malignancy presentations. Nevertheless, we also promote the report of rare cases like ours, with or without EBV viremia, as it will help share knowledge and likely decrease poor outcomes for HIV/AIDS-positive patients with unusual clinical presentations.
